# *N*-(2-Hy­droxy­eth­yl)-5-(4-meth­oxy­phen­yl)-4*H*-pyrazole-3-carboxamide

**DOI:** 10.1107/S1600536812007829

**Published:** 2012-02-29

**Authors:** Guangqian Han, Jiaguo Lv, Ju Zhu, Youjun Zhou, Defeng Wu

**Affiliations:** a Department of Pharmacognosy, School of Food Science, Fujian Agriculture and Forestry University, Fujian 350002, People’s Republic of China; b Department of Medicinal Chemistry, School of Pharmacy, Second Military Medical University, Shanghai 200433, People’s Republic of China

## Abstract

In the title compound, C_13_H_15_N_3_O_3_, the dihedral angle between the benzene and pyrazole rings is 7.7 (1)° and the O—C—C—N torsion angle of the side chain is 74.1 (2)°. In the crystal, mol­ecules are linked by O—H⋯O, N—H⋯O and N—H⋯N hydrogen bonds.

## Related literature

For the biological activities of pyrazole derivatives, see: Qi *et al.* (2011 ▶). For a related structure, see: Shi & Xie (2011 ▶).
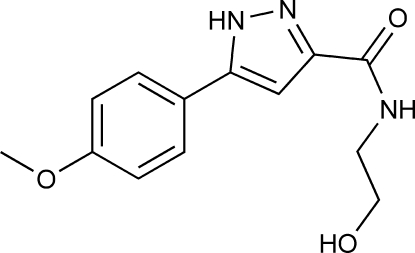


## Experimental

### 

#### Crystal data

C_13_H_15_N_3_O_3_
*M* *_r_* = 261.28
Monoclinic, 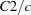

*a* = 21.82 (5) Å
*b* = 10.08 (2) Å
*c* = 12.28 (3) Å
β = 110.53 (3)°
*V* = 2531 (11) Å^3^
*Z* = 8
Mo *K*α radiation
μ = 0.10 mm^−1^
*T* = 293 K
0.20 × 0.15 × 0.06 mm

#### Data collection

Bruker SMART CCD diffractometer
Absorption correction: multi-scan (*SADABS*; Bruker, 2000 ▶) *T*_min_ = 0.980, *T*_max_ = 0.994
5292 measured reflections
2366 independent reflections
1841 reflections with *I* > 2σ(*I*)
*R*_int_ = 0.074

#### Refinement

*R*[*F* ^2^ > 2σ(*F* ^2^)] = 0.054
*wR*(*F* ^2^) = 0.156
*S* = 1.05
2366 reflections
175 parameters
H-atom parameters constrained
Δρ_max_ = 0.26 e Å^−3^
Δρ_min_ = −0.30 e Å^−3^

### 

Data collection: *SMART* (Bruker, 2000 ▶); cell refinement: *SAINT* (Bruker, 2000 ▶); data reduction: *SAINT*; program(s) used to solve structure: *SHELXS97* (Sheldrick, 2008 ▶); program(s) used to refine structure: *SHELXL97* (Sheldrick, 2008 ▶); molecular graphics: *SHELXTL* (Sheldrick, 2008 ▶); software used to prepare material for publication: *SHELXTL*.

## Supplementary Material

Crystal structure: contains datablock(s) I, global. DOI: 10.1107/S1600536812007829/hb6641sup1.cif

Structure factors: contains datablock(s) I. DOI: 10.1107/S1600536812007829/hb6641Isup2.hkl

Supplementary material file. DOI: 10.1107/S1600536812007829/hb6641Isup3.cdx

Supplementary material file. DOI: 10.1107/S1600536812007829/hb6641Isup4.cml

Additional supplementary materials:  crystallographic information; 3D view; checkCIF report

## Figures and Tables

**Table 1:** Hydrogen-bond geometry (Å, °)

*D*—H⋯*A*	*D*—H	H⋯*A*	*D*⋯*A*	*D*—H⋯*A*
O3—H3⋯O2^i^	0.82	1.88	2.668 (5)	162
N3—H3*B*⋯N2^ii^	0.86	2.54	3.318 (7)	151
N1—H1*D*⋯O3^ii^	0.86	1.90	2.739 (5)	166

Symmetry codes: (i) 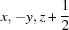; (ii) 
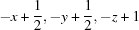
.

**Fig. 1.**
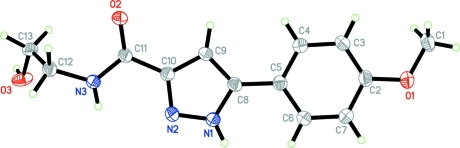
The molecular structure of the title compound, with 30% probability displacement ellipsoids for non-H atoms.

**Fig. 2.**
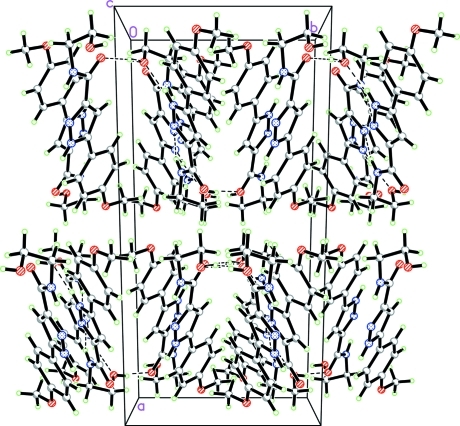
Hydrogen bonds in the title compound.

## supplementary crystallographic information

### Experimental

A mixture of diethyl oxalate (0.1 mol), 1-(4-methoxyphenyl)ethanone (0.05 mol) and sodium ethylate (400 ml 0.1 mol) were stirred for 8 h at room temperature. It was then poured into diluted acetic acid and was further stirred for 20 min and then filtered to give the yellow solid and dried. Then this dried solid and hydrazine (0.05 mol) in ethanol (200 ml) refluxed for 3.5 h and then stood for 8 h yielded the yellow solid. The solid (0.004 mol) subsequently was reacted with ethanolamine (20 ml) for 4 h at 80 °C in the presence of pyridine (20 ml). The mixture was then poured into ice cold water to afford the white solid. The compound was recrystallized from methanol as colourless slabs. Yield: 0.75 g, 70.7%. *M*. p.: 470 K.

### Refinement

All hydrogen atoms were placed in calculated positions using a riding model, with d(C—H) = 0.93 Å for aromatic, 0.97 Å for CH_2_ and 0.96 Å for CH_3_ atoms, and with *U*_iso_(H) = 1.2–1.5Ueq(C, O).

### Crystal data

**Table:**

C_13_H_15_N_3_O_3_	*D*_x_ = 1.372 Mg m^−^^3^
*M**_r_* = 261.28	Melting point: 470 K
Monoclinic, *C*2/*c*	Mo *K*α radiation, λ = 0.71073 Å
*a* = 21.82 (5) Å	Cell parameters from 925 reflections
*b* = 10.08 (2) Å	θ = 3.1–26.4°
*c* = 12.28 (3) Å	µ = 0.10 mm^−^^1^
β = 110.53 (3)°	*T* = 293 K
*V* = 2531 (11) Å^3^	Slab, colorless
*Z* = 8	0.20 × 0.15 × 0.06 mm
*F*(000) = 1104	

### Data collection

**Table:**

Bruker SMART CCD diffractometer	2366 independent reflections
Radiation source: fine-focus sealed tube	1841 reflections with *I* > 2σ(*I*)
Graphite monochromator	*R*_int_ = 0.074
phi and ω scans	θ_max_ = 25.5°, θ_min_ = 2.0°
Absorption correction: multi-scan (*SADABS*; Bruker, 2000)	*h* = −26→10
*T*_min_ = 0.980, *T*_max_ = 0.994	*k* = −12→12
5292 measured reflections	*l* = −13→14

### Refinement

**Table:**

Refinement on *F*^2^	Secondary atom site location: difference Fourier map
Least-squares matrix: full	Hydrogen site location: inferred from neighbouring sites
*R*[*F*^2^ > 2σ(*F*^2^)] = 0.054	H-atom parameters constrained
*wR*(*F*^2^) = 0.156	*w* = 1/[σ^2^(*F*_o_^2^) + (0.0917*P*)^2^ + 0.0958*P*] where*P* = (*F*_o_^2^ + 2*F*_c_^2^)/3
*S* = 1.05	(Δ/σ)_max_ < 0.001
2366 reflections	Δρ_max_ = 0.26 e Å^−^^3^
175 parameters	Δρ_min_ = −0.30 e Å^−^^3^
0 restraints	Extinction correction: *SHELXL97* (Sheldrick, 2008), Fc^*^=kFc[1+0.001xFc^2^λ^3^/sin(2θ)]^-1/4^
Primary atom site location: structure-invariant direct methods	Extinction coefficient: 0.0026 (8)

### Special details

**Table:**

Geometry. All e.s.d.'s (except the e.s.d. in the dihedral angle between two l.s. planes) are estimated using the full covariance matrix. The cell e.s.d.'s are taken into account individually in the estimation of e.s.d.'s in distances, angles and torsion angles; correlations between e.s.d.'s in cell parameters are only used when they are defined by crystal symmetry. An approximate (isotropic) treatment of cell e.s.d.'s is used for estimating e.s.d.'s involving l.s. planes.
Refinement. Refinement of *F*^2^ against ALL reflections. The weighted *R*-factor *wR* and goodness of fit *S* are based on *F*^2^, conventional *R*-factors *R* are based on *F*, with *F* set to zero for negative *F*^2^. The threshold expression of *F*^2^ > σ(*F*^2^) is used only for calculating *R*-factors(gt) *etc*. and is not relevant to the choice of reflections for refinement. *R*-factors based on *F*^2^ are statistically about twice as large as those based on *F*, and *R*- factors based on ALL data will be even larger.

### Fractional atomic coordinates and isotropic or equivalent isotropic displacement parameters (Å^2^)

**Table:**

	*x*	*y*	*z*	*U*_iso_*/*U*_eq_	
N1	0.29642 (7)	0.24543 (15)	0.29533 (13)	0.0426 (4)	
H1D	0.3331	0.2866	0.3241	0.051*	
N2	0.25083 (7)	0.24377 (15)	0.34610 (13)	0.0433 (4)	
N3	0.13697 (7)	0.21072 (15)	0.39238 (13)	0.0414 (4)	
H3B	0.1720	0.2410	0.4441	0.050*	
O1	0.44500 (7)	0.12084 (15)	−0.06540 (14)	0.0593 (5)	
O2	0.09398 (6)	0.09459 (14)	0.22772 (11)	0.0526 (4)	
O3	0.09522 (7)	0.10663 (15)	0.58747 (12)	0.0575 (4)	
H3	0.0904	0.0361	0.6168	0.086*	
C1	0.42987 (12)	0.0298 (2)	−0.15960 (19)	0.0621 (6)	
H1A	0.4244	−0.0573	−0.1329	0.093*	
H1B	0.4649	0.0286	−0.1898	0.093*	
H1C	0.3901	0.0567	−0.2197	0.093*	
C2	0.40166 (9)	0.13059 (18)	−0.00812 (17)	0.0435 (5)	
C3	0.34387 (9)	0.06118 (19)	−0.03558 (17)	0.0470 (5)	
H3A	0.3312	0.0034	−0.0986	0.056*	
C4	0.30463 (9)	0.0776 (2)	0.03073 (18)	0.0455 (5)	
H4	0.2660	0.0292	0.0120	0.055*	
C5	0.32117 (8)	0.16408 (18)	0.12430 (15)	0.0387 (4)	
C6	0.37895 (10)	0.23460 (19)	0.14879 (18)	0.0481 (5)	
H6	0.3909	0.2949	0.2100	0.058*	
C7	0.41911 (10)	0.2177 (2)	0.08479 (19)	0.0512 (6)	
H7	0.4581	0.2649	0.1041	0.061*	
C8	0.27897 (9)	0.17627 (17)	0.19481 (15)	0.0382 (4)	
C9	0.21809 (9)	0.12518 (19)	0.17982 (16)	0.0416 (5)	
H9	0.1924	0.0720	0.1192	0.050*	
C10	0.20311 (8)	0.17037 (18)	0.27537 (16)	0.0386 (4)	
C11	0.14091 (8)	0.15331 (17)	0.29704 (15)	0.0377 (4)	
C12	0.07505 (9)	0.22326 (18)	0.41093 (16)	0.0400 (5)	
H12A	0.0407	0.2374	0.3362	0.048*	
H12B	0.0769	0.3015	0.4579	0.048*	
C13	0.05641 (9)	0.10679 (19)	0.46890 (16)	0.0426 (5)	
H13A	0.0105	0.1126	0.4600	0.051*	
H13B	0.0630	0.0250	0.4329	0.051*	

### Atomic displacement parameters (Å^2^)

**Table:**

	*U*^11^	*U*^22^	*U*^33^	*U*^12^	*U*^13^	*U*^23^
N1	0.0394 (8)	0.0550 (10)	0.0340 (9)	−0.0095 (7)	0.0135 (7)	−0.0065 (7)
N2	0.0441 (9)	0.0540 (10)	0.0343 (9)	−0.0066 (7)	0.0169 (7)	−0.0064 (7)
N3	0.0376 (8)	0.0561 (10)	0.0301 (8)	−0.0080 (7)	0.0114 (7)	−0.0086 (7)
O1	0.0660 (10)	0.0645 (10)	0.0627 (10)	−0.0145 (7)	0.0417 (9)	−0.0168 (7)
O2	0.0388 (8)	0.0742 (10)	0.0420 (8)	−0.0095 (6)	0.0107 (6)	−0.0206 (7)
O3	0.0585 (9)	0.0622 (10)	0.0378 (8)	−0.0229 (7)	−0.0006 (7)	0.0077 (6)
C1	0.0806 (16)	0.0644 (14)	0.0562 (14)	−0.0077 (12)	0.0429 (13)	−0.0116 (11)
C2	0.0514 (11)	0.0425 (10)	0.0417 (11)	−0.0008 (8)	0.0229 (9)	0.0007 (8)
C3	0.0550 (12)	0.0480 (11)	0.0410 (11)	−0.0055 (9)	0.0205 (10)	−0.0091 (9)
C4	0.0432 (10)	0.0507 (11)	0.0427 (11)	−0.0080 (8)	0.0152 (9)	−0.0054 (9)
C5	0.0413 (10)	0.0416 (10)	0.0336 (10)	0.0009 (8)	0.0134 (8)	0.0027 (8)
C6	0.0537 (12)	0.0507 (11)	0.0441 (11)	−0.0116 (9)	0.0225 (10)	−0.0121 (9)
C7	0.0520 (12)	0.0549 (12)	0.0527 (13)	−0.0147 (9)	0.0257 (10)	−0.0094 (10)
C8	0.0418 (10)	0.0413 (9)	0.0301 (10)	−0.0004 (8)	0.0108 (8)	−0.0003 (7)
C9	0.0403 (10)	0.0514 (11)	0.0318 (10)	−0.0060 (8)	0.0110 (8)	−0.0068 (8)
C10	0.0367 (9)	0.0458 (10)	0.0320 (9)	−0.0017 (7)	0.0102 (8)	−0.0015 (8)
C11	0.0379 (10)	0.0443 (10)	0.0298 (9)	−0.0006 (8)	0.0106 (8)	−0.0013 (7)
C12	0.0388 (10)	0.0486 (11)	0.0320 (10)	0.0018 (8)	0.0118 (8)	−0.0009 (8)
C13	0.0390 (10)	0.0499 (11)	0.0355 (10)	−0.0082 (8)	0.0087 (8)	−0.0044 (8)

### Geometric parameters (Å, º)

**Table:**

N1—N2	1.347 (3)	C3—H3A	0.9300
N1—C8	1.351 (3)	C4—C5	1.386 (4)
N1—H1D	0.8600	C4—H4	0.9300
N2—C10	1.324 (3)	C5—C6	1.385 (4)
N3—C11	1.336 (4)	C5—C8	1.474 (3)
N3—C12	1.452 (4)	C6—C7	1.378 (4)
N3—H3B	0.8600	C6—H6	0.9300
O1—C2	1.366 (3)	C7—H7	0.9300
O1—C1	1.422 (4)	C8—C9	1.376 (4)
O2—C11	1.230 (3)	C9—C10	1.400 (4)
O3—C13	1.405 (4)	C9—H9	0.9300
O3—H3	0.8200	C10—C11	1.482 (4)
C1—H1A	0.9600	C12—C13	1.502 (4)
C1—H1B	0.9600	C12—H12A	0.9700
C1—H1C	0.9600	C12—H12B	0.9700
C2—C3	1.377 (4)	C13—H13A	0.9700
C2—C7	1.383 (4)	C13—H13B	0.9700
C3—C4	1.383 (3)		
			
N2—N1—C8	113.55 (19)	C5—C6—H6	119.2
N2—N1—H1D	123.2	C6—C7—C2	120.2 (2)
C8—N1—H1D	123.2	C6—C7—H7	119.9
C10—N2—N1	104.0 (2)	C2—C7—H7	119.9
C11—N3—C12	121.65 (15)	N1—C8—C9	105.35 (16)
C11—N3—H3B	119.2	N1—C8—C5	123.1 (2)
C12—N3—H3B	119.2	C9—C8—C5	131.5 (2)
C2—O1—C1	117.42 (19)	C8—C9—C10	105.33 (19)
C13—O3—H3	109.5	C8—C9—H9	127.3
O1—C1—H1A	109.5	C10—C9—H9	127.3
O1—C1—H1B	109.5	N2—C10—C9	111.8 (2)
H1A—C1—H1B	109.5	N2—C10—C11	120.4 (2)
O1—C1—H1C	109.5	C9—C10—C11	127.62 (17)
H1A—C1—H1C	109.5	O2—C11—N3	121.4 (2)
H1B—C1—H1C	109.5	O2—C11—C10	121.6 (2)
O1—C2—C3	125.3 (2)	N3—C11—C10	116.86 (16)
O1—C2—C7	115.5 (2)	N3—C12—C13	115.33 (17)
C3—C2—C7	119.21 (18)	N3—C12—H12A	108.4
C2—C3—C4	119.9 (2)	C13—C12—H12A	108.4
C2—C3—H3A	120.1	N3—C12—H12B	108.4
C4—C3—H3A	120.1	C13—C12—H12B	108.4
C3—C4—C5	121.9 (2)	H12A—C12—H12B	107.5
C3—C4—H4	119.0	O3—C13—C12	109.12 (19)
C5—C4—H4	119.0	O3—C13—H13A	109.9
C6—C5—C4	117.09 (18)	C12—C13—H13A	109.9
C6—C5—C8	122.5 (2)	O3—C13—H13B	109.9
C4—C5—C8	120.3 (2)	C12—C13—H13B	109.9
C7—C6—C5	121.7 (2)	H13A—C13—H13B	108.3
C7—C6—H6	119.2		

### Hydrogen-bond geometry (Å, º)

**Table:**

*D*—H···*A*	*D*—H	H···*A*	*D*···*A*	*D*—H···*A*
O3—H3···O2^i^	0.82	1.88	2.668 (5)	162
N3—H3*B*···N2^ii^	0.86	2.54	3.318 (7)	151
N1—H1*D*···O3^ii^	0.86	1.90	2.739 (5)	166

Symmetry codes: (i) *x*, −*y*, *z*+1/2; (ii) −*x*+1/2, −*y*+1/2, −*z*+1.
